# A maize architect: An epiallele of a PfkB-type carbohydrate kinase affects plant growth and development

**DOI:** 10.1093/plcell/koaf025

**Published:** 2025-02-03

**Authors:** Meenu Singla-Rastogi

**Affiliations:** Assistant Features Editor, The Plant Cell, American Society of Plant Biologists; Department of Biology, Indiana University, Bloomington, IN 47405, USA

The maize (*Zea mays*) genome is known for its complexity, largely due to the high content of transposable elements (TEs), which contribute significantly to both its size and structural variation (Schnable et al. 2009). Transposons, also known as “jumping genes,” are DNA sequences capable of moving from one location to another within the genome. In maize, transposons account for nearly 85% of the genome—substantially more than in many other organisms. The activity of these transposons is tightly regulated, often through epigenetic mechanisms that suppress their movement. These mechanisms include DNA methylation and histone modifications, which maintain genome stability by silencing transposon activity (Slotkin and Martienssen 2007). However, when these regulatory processes are disrupted, transposons become active again, potentially leading to undesired mutations or genomic instability. Interestingly, controlled activation of transposons has allowed researchers to explore genetic diversity and discover new traits, contributing to the development of maize varieties with higher yields, enhanced stress tolerance, and improved quality.

Plant architecture plays a significant role in determining the grain yield in maize. The way a maize plant is structured—how it develops leaves, branches, ears, and roots—directly influences its ability to capture light, absorb nutrients, and support grain production. In a new work, **Ruonan Li and colleagues (Li et al. 2025)** discovered and characterized a naturally occurring epiallele (epigenetic variant) of a gene encoding a phosphofructokinase B (PfkB)-type carbohydrate kinase, which they named *Plant architecture 1* or *par1*. While constructing a recombinant inbred line population using maize inbred Zheng58 and the landrace BDB, the authors identified a spontaneous mutant with pleiotropic phenotype, exhibiting reduced plant height, shorter and narrower leaves, and larger leaf angle than the wild-type plants. Further genetic analysis revealed that the *par1* epiallele was caused by a *de novo* intronic insertion of a CACTA transposon, a member of Class II transposons known for their “cut-and-paste” mechanism of transposition (Wicker et al. 2007). To confirm the role of the CACTA transposon in the *par1* phenotype, the authors conducted genotyping of the F_2_ population. Surprisingly, even though all the mutant plants were homozygous for the insertion, only about one-half of the plants with homozygous insertion exhibited the mutant phenotype. As a result, the authors categorized the plants with homozygous insertion into two groups: *epinormal* (with a wild-type phenotype) and *epimutant* (with the mutant phenotype).

This phenomenon of differing phenotypes despite identical genetic sequences is akin to what is observed in identical human twins, where subtle phenotypic differences arise due to epigenetic divergence (Wong et al. 2005). It has been shown that CACTA transposons contribute to phenotypic diversity in maize, with their effects modulated by DNA methylation patterns (Wittmeyer et al. 2018). Through bulk segregant RNA-seq and RT-PCR analyses, the researchers confirmed that the *par1* gene was normally expressed in epinormal plants, but its expression was significantly reduced in epimutants. These differences in expression were correlated with the phenotypic differences between the two groups. To identify the underlying epigenetic factors, the researchers employed whole-genome bisulfite sequencing and examined DNA methylation levels around the CACTA insertion site. The analysis revealed high DNA methylation at the edges of the transposon in epinormal plants and low methylation levels in epimutants. This finding supports the idea that DNA methylation influences gene expression, as increased CHH (where H corresponds to A, T, or C) methylation upstream of a gene is typically associated with higher gene expression levels (Li et al. 2015).

Further biochemical analysis revealed that the *par1* epiallele encodes PAR1, a PfkB-type carbohydrate kinase capable of phosphorylating several nucleosides (inosine, uridine, adenosine, cytidine, and guanosine) into nucleoside monophosphates. This process is part of a nucleotide salvage pathway, which converts nucleosides or nucleobases into their phosphorylated forms. While the role of nucleoside kinases in Arabidopsis has been well studied (Witte and Herde 2020), there are fewer reports on their function in other plants. This study provides valuable insights into the role of PfkB-like proteins in regulating the abundance of nucleosides and nucleotides, which are crucial for plant development.

To investigate whether the loss of PAR1 kinase activity is responsible for the observed stunted phenotype in the mutant, the researchers treated the mutant plants with different nucleotides *in vitro*. The treatment partially rescued the stunted growth, suggesting that the kinase activity of PAR1 is at least partially responsible for the mutant phenotype. Additionally, RNA-seq analysis comparing the *par1* mutants with wild-type plants revealed significant alterations in the jasmonic acid (JA) biosynthetic and signaling pathways. This misregulation of the JA pathway was confirmed by high levels of JA in the mutant plants.

The findings of this study suggest a connection between nucleotide metabolism and the regulation of plant architecture, with particular emphasis on the role of transposon-mediated epigenetic regulation of gene expression. Notably, this research uncovers a molecular mechanism through which nucleotide and JA metabolism influence plant development (Figure). While DNA methylation is typically associated with gene silencing (Xu et al. 2022), this study sheds light on the complex dynamics between high DNA methylation and high gene expression, providing insights into previously unexplained phenomena. However, the precise relationship between nucleotide metabolism and JA metabolism still remains an open question that warrants further investigation.

**Figure.**
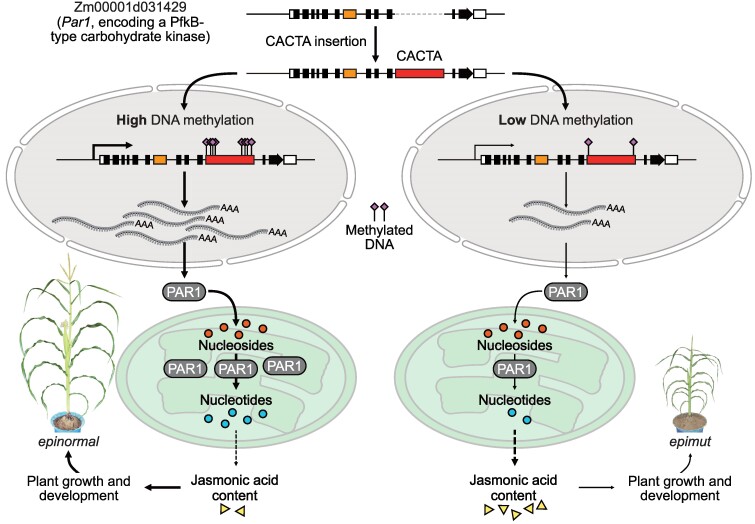
Working model for the role of the CACTA transposon in regulating gene expression and plant development. When the CACTA transposon contains high levels of DNA methylation (left), *par1* is expressed normally. The translated PAR1 protein is localized to the chloroplast, where PAR1 catalyzes the conversion of nucleosides to nucleoside monophosphates. In this case, the JA content of the plant is normal, and the plant develops normally. When the CACTA transposon contains low levels of DNA methylation (right), *par1* is barely expressed. After translation, only a small amount of PAR1 protein enters the chloroplast, leading to a decrease in nucleotide contents in the chloroplast. This, in turn, leads to an increase in JA content, resulting in stunted plant growth and delayed development. Reprinted from Li et al. (2025), Figure 9.

## Data Availability

No new data were generated or analyzed in support of this article.

## Dive Curated Terms

The following phenotypic, genotypic, and functional terms are of significance to the work described in this paper:

PAR1 Gramene: Zm00001d031429
PAR1 Araport: Zm00001d031429
